# Association between estimated glucose disposal rate and female infertility: a cross-sectional study

**DOI:** 10.3389/fendo.2024.1474738

**Published:** 2024-11-12

**Authors:** Meng Li, Lisong Zhang, Xiaoyu Li, Weisheng Yan

**Affiliations:** Department of Gynecology, Fuxing Hospital, Capital Medical University, Beijing, China

**Keywords:** estimated glucose disposal rate, female fertility, infertility, insulin resistance, NHANES

## Abstract

**Background:**

Insulin resistance (IR) can lead to infertility in women. The primary objective of this research was to examine how estimated glucose disposal rate (eGDR) correlates with infertility in women, assessing its validity as an indicator of IR.

**Methods:**

Data from the National Health and Nutrition Examination Survey spanning 2013 to 2018 were analyzed in this study. In order to investigate the correlation between eGDR and the prevalence of female infertility, this study used a combination of weighted multivariate regression analysis, restricted cubic spline (RCS) analysis, subgroup analyses, sensitive analysis, and receiver operating characteristic (ROC) curves.

**Results:**

This study enrolled 2541 women, with an average age of (32.52 ± 0.23) years. The overall infertility rate was 14.27%. A negative relationship was observed between eGDR levels and female infertility. Each increment of one unit in eGDR was linked to a 14% reduction in infertility incidence (OR = 0.86, 95% CI 0.80–0.94). RCS analysis revealed a nonlinear, inverse correlation between eGDR and female infertility. Subgroup analyses indicated that age influenced the association between eGDR and female infertility. The ROC curve suggested that eGDR was significantly better than HOMA-IR in predicting infertility [eGDR: 0.632 (95% CI: 0.603, 0.660) vs. HOMA-IR: 0.543 (95% CI: 0.514, 0.572)].

**Conclusion:**

There was an observed association where lower eGDR levels were linked with higher rates of female infertility. These results emphasize the significance of implementing measures to manage IR to protect women’s reproductive health.

## 1 Introduction

Infertility is defined as the inability to achieve conception following twelve months or more of consistent, unprotected sexual intercourse without contraception (1). Globally, approximately 10% to 15% of couples in their reproductive years’ experience infertility (2). The incidence of infertility has continued to rise in recent years as a result of environmental, social, and lifestyle changes, and infertility has been recognized by the World Health Organization (WHO) as a substantial public health issue of global concern (3). Infertility creates a serious emotional stress and financial burden on the couple. Recent reports indicate that women with infertility have a significantly elevated risk of perinatal depression, even after successful pregnancy following treatment (4). The U. S. Centers for Disease Control and Prevention (CDC) has recommended focusing on the prevention, detection, and management of infertility as a priority (5).

The causes of infertility are complex and include male infertility, ovulation disorders, tubal factors and unexplained infertility. Age, obesity, smoking, alcohol consumption, and past history may be associated with female infertility. At the same time, abnormalities of glucose and lipid metabolism are common in infertile women (6). Insulin resistance (IR) may result in endocrine disruptions, impacting follicular development, oocyte quality, and ovulation patterns in women. These observations underscore IR’s significant role in female infertility (7). Polycystic ovary syndrome (PCOS) has garnered considerable focus regarding the association between IR and infertility. However, many studies have substantiated the presence of IR as a distinct factor in infertile women, may not be associated with PCOS (7, 8). This pathology is prevalent among individuals experiencing fertility challenges. The hyperinsulinemic-euglycemic clamp (HIEC) is regarded as the gold standard for assessing IR, but its clinical application is constrained by its invasive nature and cost implications (9). The homeostasis model assessment of insulin resistance (HOMA-IR) is commonly employed in clinical settings, but it has certain limitations for patients undergoing insulin therapy (10). The estimated glucose disposal rate (eGDR) was originally designed to measure IR in type 1 diabetes (T1D), utilizing parameters such as waist circumference, hypertension, and glycosylated hemoglobin A (HbA1c) for its calculation (11). This approach demonstrates superior accuracy and is consequently highly advantageous for assessing IR across extensive cohorts of patients (11, 12). Studies also indicated that reduced eGDR correlated with heightened susceptibility to conditions linked to IR, including nonalcoholic fatty liver disease, cardiovascular disorders, and incidents of acute ischemic stroke, among others (13–15).

The relationship between eGDR and female fertility has not been definitively established. This study aimed to investigate the relationship between eGDR levels and female infertility using data from the National Health and Nutrition Examination Survey (NHANES), aiming to offer new insights for academic research on the prevention and management of women’s fertility.

## 2 Methods

### 2.1 Survey description and participants

The dataset utilized in this study was sourced from NHANES, a prominent national survey managed by the National Center for Health Statistics (NCHS). NHANES performs thorough evaluations to evaluate the nutritional and health well-being of the American populace through a biennial, multistage, randomized sampling approach. This survey encompasses interviews, physical examinations, and laboratory analyses. Data are derived from direct measurements (e.g., blood samples, anthropometric data) and self-reported surveys (e.g., dietary intake, health behaviors). NHANES aims to furnish extensive insights into the health and nutritional profiles of the American populace. These insights are crucial for evaluating disease prevalence, investigating health risk factors, and elucidating epidemiological trends. Every participant signed an informed consent, and approval for the entire investigative process was granted by the Ethics Committee of the NCHS.

**Figure 1:**
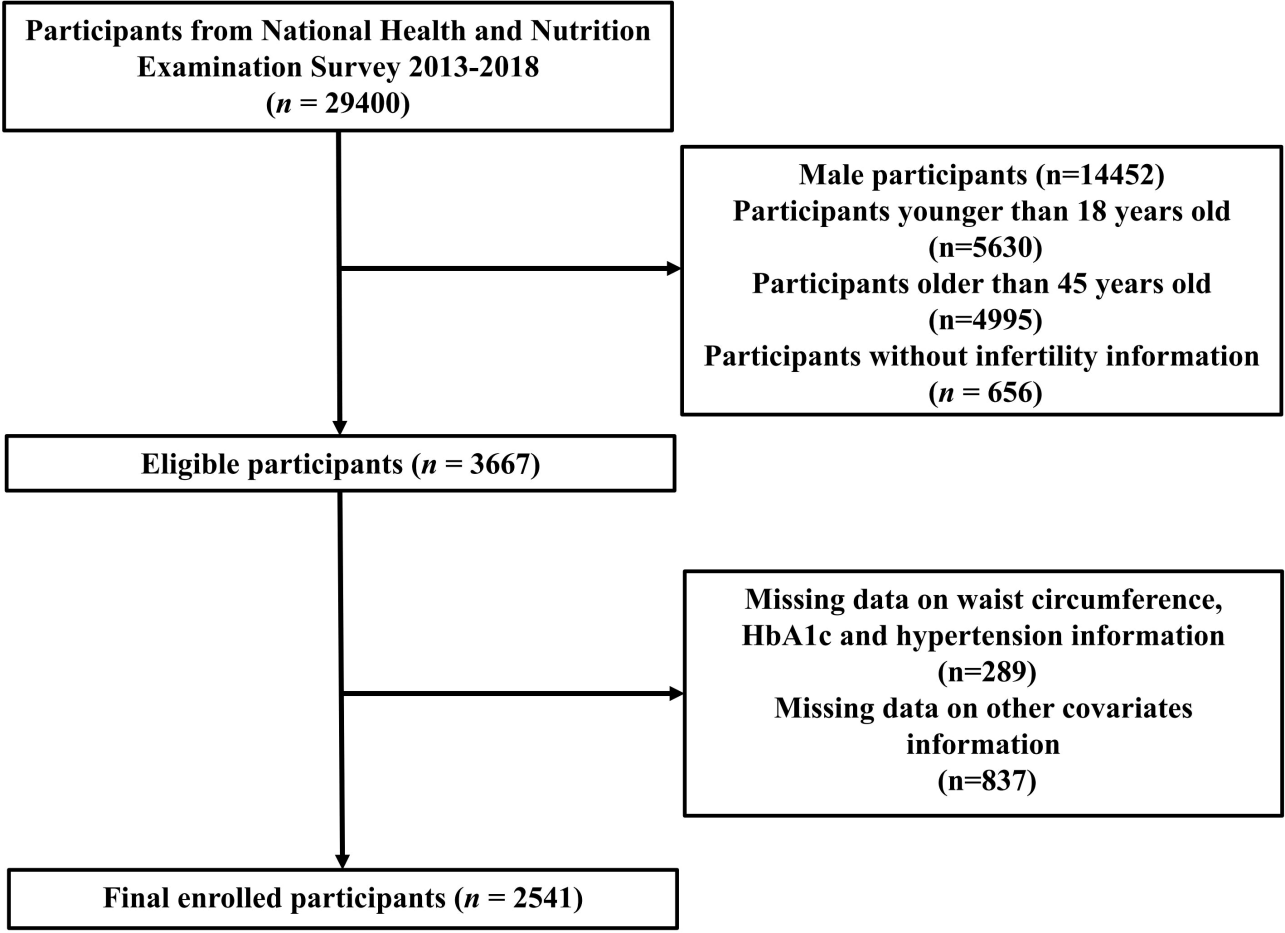
Flowchart.

This study analyzed NHANES data from the years 2013 to 2018. Initially, the study encompassed a cohort of 29,400 participants. However, we excluded 14,452 males, 10,625 participants aged < 18 or > 45 years, 656 participants without infertility questionnaire data, 289 participants without complete eGDR data, and 837 participants missing data on covariates information. Ultimately, the final analysis involved 2,541 participants in total (**Figure 1**).

### 2.2 Independent variables

The formula for calculating eGDR in mg/kg/min was derived from established literature and was expressed as follows: 21.158 + [-0.09 * waist circumference (WC)] + [-3.407 * hypertension (HP) (yes = 1/no = 0)] + (-0.551 * HbA1c) (16–18). To accurately measure WC, start by extending a horizontal line from the outermost point of the right ilium and marking the right mid-axillary line. Position a measuring tape precisely at the intersection of these markers and record the measurement in centimeters (cm). HP was determined based on documented history, systolic blood pressure ≥ 140 mmHg, diastolic blood pressure ≥ 90 mmHg, or use of antihypertensive medications during the study period. HbA1c levels were quantified using boronate-affinity chromatography in conjunction with high-performance liquid chromatography, with HbA1c representing the percentage of glycated hemoglobin. The HOMA-IR index was calculated as follows: HOMA-IR = fasting glucose (mmol/L) × fasting insulin (µU/mL)/22.5 (19).

### 2.3 Assessment of fertility

Referring to previous studies, infertility was assessed by self-reporting of women on the Reproductive Health Questionnaire 074 (20–23). The participants were asked, “Have you ever tried to get pregnant for at least one year without getting pregnant?” Reproductive status was classified as infertile for women who responded “yes”.

### 2.4 Covariates

We additionally investigated potential variables that could influence the correlation between eGDR and female infertility. Demographic variables included age, race, education level, family income-to-poverty ratio (PIR), marital status. Those classified as smokers had a documented history of smoking over 100 cigarettes in their lifetime. Alcohol use was characterized by consuming a minimum of 12 alcoholic beverages in the year prior to the survey. Anthropometric indicators included body mass index (BMI) and WC. Information regarding the regularity of menstrual cycles and history of pelvic inflammatory disease (PID) was obtained through reproductive questionnaires. Information on adrenocortical insufficiency and sex hormonal dysfunctions, including polycystic ovarian syndrome, premature menopause and ovarian dysfunction was sourced from the prescription drug panel. Chronic diseases are including HP and diabetes mellitus (DM). The diagnostic criteria for DM included physician-diagnosed diabetes, glycated hemoglobin (HbA1c) levels greater than 6.5%, fasting blood glucose levels exceeding 7.0 mmol/L, random blood glucose levels of 11.1 mmol/L or higher, results of 11.1 mmol/L or higher on a 2-hour oral glucose tolerance test, and the administration of diabetes medications or insulin. Laboratory tests following standardized procedures were utilized to measure hemoglobin, HbA1c, and total cholesterol (TC).

### 2.5 Statistical analysis

In analyzing this study, the intricate sampling framework of NHANES necessitated the utilization of sample weights. These weights were crucially incorporated to ensure the statistical integrity and representativeness of the findings. Within this study cohort, an analysis of demographic characteristics and measured parameters was conducted, categorizing these indicators into groups categorized by whether infertility was present or not. Mean ± standard error of the mean (SEM) was utilized to describe continuous variables, and t-tests were employed for intergroup comparisons. Frequency distributions [n (%)] were employed to describe categorical variables, with group comparisons conducted using the chi-square test. Weighted multivariate logistic regression analysis was employed to investigate the relationship between eGDR and female infertility, with results reported as odds ratios (OR) and their corresponding 95% confidence intervals (CI). Three statistical models of multivariate logistic regression were employed: Model 1 remained unadjusted, Model 2 adjusted for age, race, and education levels, and Model 3 adjusted for age, race/ethnicity, education levels, PIR, marital status, smoking, drinking, hemoglobin, TC, DM, regular period, PID, adrenocortical insufficiency and sex hormone dysfunction. The relationship between eGDR and infertility prevalence was explored using the restricted cubic spline (RCS) analysis. Analyses were stratified by age, race, education level, BMI, PIR, menstrual cycle characteristics, pelvic infection status, and DM history for subgroup evaluation. NHANES employs advanced sampling methods to enhance the accuracy and relevance of its findings. Nonetheless, differences can arise between weighted and unweighted analyses. To address this, we performed a sensitivity analysis using unweighted regression to confirm our results. Finally, the predictive ability of HOMA-IR and eGDR for infertility was compared by means of receiver operating characteristic (ROC) curves and their respective areas under the curve (AUCs). Differences between AUCs were compared by means of z-tests. Data analysis was performed utilizing R software version 4.1.6, with a significance level of *P* < 0.05.

## 3 Results

### 3.1 Baseline characteristics

The study cohort consisted of females with a mean age of 32.52 ± 0.23 years. The overall prevalence of infertility was 14.27%. Infertile women exhibited advanced age, higher BMI and WC, elevated HbA1c and TC levels, along with a higher incidence of HP, DM, PID and adrenocortical insufficiency (all *P* < 0.05). Importantly, infertile women showed significantly lower eGDR values (8.11 ± 0.23 vs 9.25 ± 0.08, *P <*0.001), suggesting that they have reduced insulin sensitivity (**Table 1**). The demographic and clinical characteristics of the cohort, organized into eGDR quantiles, were detailed in **Supplementary Table S1**. As eGDR values decreased, there was a corresponding increase in the prevalence of infertility (*P <*0.001).

**Table 1:** Clinical characteristics of study population.

Variables	Overall	Non-infertility	Infertility	*P* value
Age, years	32.52 ± 0.23	32.04 ± 0.23	35.39 ± 0.52	<0.001***
Race/ethnicity, %				0.08
White	58.31 (49.96,66.66)	57.30 (52.50,62.10)	64.41 (56.92,71.90)	
Black	12.47 (10.07,14.88)	12.63 (9.84,15.42)	11.56 (8.17,14.95)	
Mexican	11.30 (8.51,14.09)	11.47 (8.60,14.33)	10.32 (5.83,14.80)	
Others	17.91 (15.70,20.13)	18.61 (16.29,20.94)	13.72 (9.44,17.99)	
Education levels, %				0.47
Less than high school	9.84 (8.26,11.42)	10.14 (8.44,11.83)	8.08 (5.24,10.92)	
High school or equivalent	18.30 (15.65,20.95)	17.99 (15.43,20.55)	20.17 (13.50,26.84)	
College or above	71.86 (64.32,79.39)	71.87 (68.23,75.52)	71.75 (64.70,78.79)	
PIR, %				0.42
≤1.30	27.68 (25.21,30.15)	28.26 (25.36,31.17)	24.20 (18.53,29.87)	
1.31–3.49	36.88 (33.00,40.76)	36.62 (33.95,39.30)	38.40 (32.07,44.74)	
≥3.50	35.44 (30.03,40.84)	35.11 (31.54,38.69)	37.39 (29.95,44.83)	
Marital status, %				<0.001***
Married	59.73 (53.70,65.77)	57.23 (53.98,60.48)	74.79 (69.49,80.10)	
Never married	29.74 (26.18,33.29)	32.35 (29.13,35.57)	14.02 (10.61,17.44)	
Divorced	10.53 (8.57,12.49)	10.42 (8.41,12.43)	11.19 (6.53,15.85)	
BMI, kg/m^2^	29.37 ± 0.26	28.98 ± 0.28	31.70 ± 0.75	0.002**
Smoking, %				0.06
No	67.57 (62.12,73.02)	68.40 (65.56,71.24)	62.60 (57.09,68.12)	
Yes	32.43 (28.38,36.48)	31.60 (28.76,34.44)	37.40 (31.88,42.91)	
Drinking, %				0.69
No	17.33 (13.96,20.70)	17.49 (14.23,20.75)	16.37 (11.02,21.73)	
Yes	82.67 (75.36,89.98)	82.51 (79.25,85.77)	83.63 (78.27,88.98)	
Hypertension, %				<0.001***
No	85.00 (77.64,92.36)	86.55 (84.85,88.25)	75.70 (69.60,81.81)	
Yes	15.00 (13.22,16.78)	13.45 (11.75,15.15)	24.30 (18.19,30.40)	
DM, %				0.01*
No	93.37 (85.70,101.04)	93.94 (92.77,95.10)	89.96 (87.11,92.82)	
Yes	6.63 (5.56, 7.70)	6.06 (4.90, 7.23)	10.04 (7.18,12.89)	
Regular period, %				0.08
No	11.69 (9.91,13.48)	11.10 (9.47,12.72)	15.28 (10.72,19.84)	
Yes	88.31 (81.32,95.30)	88.90 (87.28,90.53)	84.72 (80.16,89.28)	
PID, %				<0.001***
No	95.51 (87.83,103.20)	96.22 (95.21,97.23)	91.28 (87.55,95.01)	
Yes	4.49 (3.35, 5.62)	3.78 (2.77, 4.79)	8.72 (4.99,12.45)	
Adrenocortical insufficiency, %				0.02*
No	99.98 (92.10,107.86)	100.00 (100.00,100.00)	99.83 (99.51,100.16)	
Yes	0.02 (0.01, 0.07)	0.00 (0.00,0.00)	0.17 (0.01,0.49)	
Sex hormonal dysfunctions, %				0.92
No	99.82 (91.95,107.68)	99.82 (99.60,100.03)	99.80 (99.40,100.20)	
Yes	0.18 (0.01, 0.38)	0.18 (0.03,0.40)	0.20 (0.10,0.60)	
waist circumference,cm	95.72 ± 0.59	94.64 ± 0.63	102.20 ± 1.58	<0.001***
HbA1c, %	5.34 ± 0.01	5.32 ± 0.02	5.48 ± 0.04	0.002**
TC, mmol/L	4.66 ± 0.03	4.64 ± 0.03	4.79 ± 0.07	0.02*
HOMA-IR	3.19 ± 0.12	3.13 ± 0.13	3.52 ± 0.30	0.23
eGDR	9.09 ± 0.08	9.25 ± 0.08	8.11 ± 0.23	<0.001***

Continuous data were presented as the mean ± SEM, category data were presented as the proportion and 95% confidence interval. SEM, Standard Error of the Mean; HOMA-IR, homeostasis model assessment of insulin resistance; PIR, poverty income ratio; BMI, body mass index; DM, diabetes mellitus; PID, pelvic inflammatory disease; HbA1c, glycosylated hemoglobin; eGDR, estimated glucose disposal rate; TC, total cholesterol; ***P value<0.001, **P value<0.01, *P value<0.05.

### 3.2 Association between eGDR and female infertility

**Figure 2:**
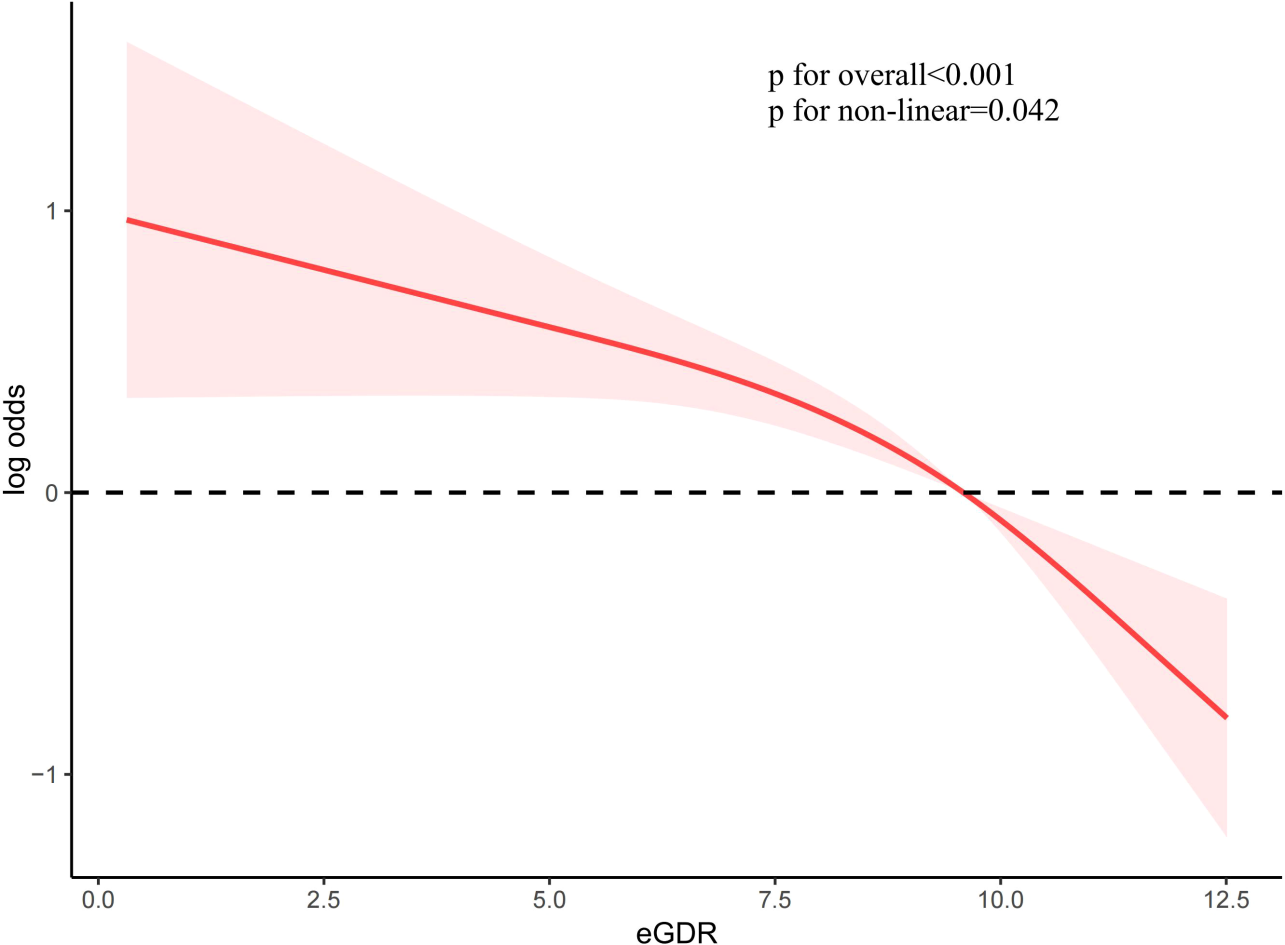
Restricted cubic spline of eGDR and infertility.

To investigate the association between eGDR and female infertility, a weighted logistic regression analysis was conducted. Across all three statistical models, the analysis consistently demonstrated an inverse relationship between eGDR levels and the prevalence of infertility. In the fully adjusted model, each incremental unit rise in eGDR was associated with a 14% reduction in the likelihood of infertility occurrence (OR = 0.86; 95% CI 0.80–0.94, *P <*0.001). When eGDR was grouped into quartiles, quartile 4 exhibited a significant 70% lower prevalence of infertility compared to quartile 1 (*P <*0.001) (**Table 2**). Analysis of RCS revealed a notable correlation between eGDR and infertility prevalence, indicating a statistically significant non-linear pattern (*P* < 0.05). As eGDR levels increased, a consistent decrease was noted in the incidence of infertility (**Figure 2**).

**Table 2:** Weighted logistic regression analysis of HOMA-IR and eGDR in relation to infertility.

	Model 1		Model 2		Model 3	
	OR (95% CI)	*P* value	OR (95% CI)	*P* value	OR (95% CI)	*P* value
HOMA-IR	1.02 (0.99,1.05)	0.16	1.02 (0.99,1.06)	0.21	1.01 (0.96,1.07)	0.61
Continuous eGDR	0.85 (0.80,0.90)	<0.001***	0.87 (0.81,0.93)	<0.001***	0.86 (0.80,0.94)	<0.001***
eGDR-Q1	Reference	–	Reference	–	Reference	–
eGDR-Q2	0.62 (0.41,0.94)	0.02*	0.65 (0.42,1.01)	0.06	0.68 (0.43, 1.07)	0.09
eGDR-Q3	0.52 (0.32,0.84)	0.01*	0.56 (0.34,0.93)	0.03*	0.58 (0.34, 0.99)	0.05
eGDR-Q4	0.25 (0.15,0.40)	<0.001***	0.30 (0.18,0.53)	<0.001***	0.30 (0.17, 0.54)	<0.001***
*P* for trend		<0.001***		<0.001***		<0.001***

Data are presented as OR (95% CI). Model 1 remained unadjusted. Model 2 adjusted for age, race/ethnicity and education levels. Model 3 adjusted for age, race/ethnicity, education levels, poverty income ratio, marital status, smoking, drinking, hemoglobin, TC, DM, regular period and PID, adrenocortical insufficiency and sex hormone dysfunction. ***P value<0.001, *P value<0.05.

### 3.3 Subgroup analysis

**Figure 3:**
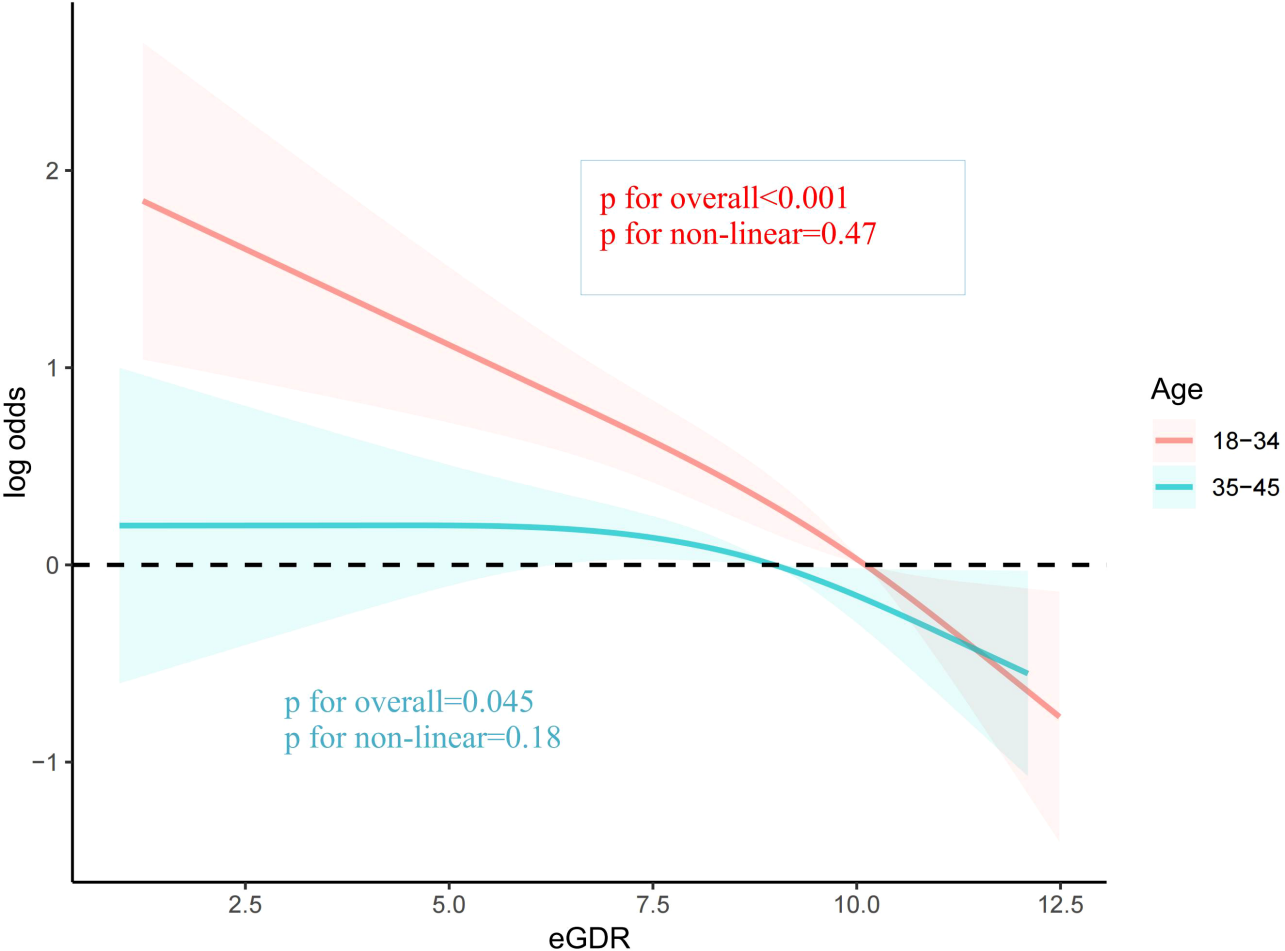
The association between eGDR and infertility stratified by age.

To explore the potential influence of covariates on the relationship between eGDR and female infertility prevalence, subgroup analyses were conducted. Upon comprehensive adjustment for all covariates, the analyses revealed that the association between eGDR and infertility prevalence was significantly influenced by age (*P* for interaction < 0.001). Specifically, the inverse relationship between eGDR and infertility was notably stronger among women under 35 years of age (OR = 0.78, 95% CI 0.72-0.84). In the remaining subgroups analyzed, no significant interactions were observed (**Table 3**). The RCS analysis also indicated that eGDR conferred more pronounced benefits among women aged under 35 years (**Figure 3**).

**Table 3:** Subgroup analysis for the association between the eGDR and infertility.

Variable name	Non-infertility	Infertility	*P* value	*P* for interaction
Age				<0.001***
18-34 years	ref	0.78(0.72,0.84)	<0.001	
35-45 years	ref	0.94(0.87,1.02)	0.14	
BMI				0.15
≤30	ref	0.81(0.74,0.89)	<0.001	
>30	ref	0.88(0.81,0.95)	0.002	
Race				0.55
White	ref	0.83(0.77,0.90)	<0.001	
Black	ref	0.88(0.80,0.97)	0.01	
Mexican American	ref	0.82(0.73,0.92)	0.002	
Others	ref	0.88(0.78,0.99)	0.04	
Education levels				0.43
less than high school	ref	0.78(0.68,0.90)	<0.001	
high school or equivalent	ref	0.83(0.75,0.93)	0.001	
college or above	ref	0.86(0.80,0.92)	<0.001	
Marital status				0.30
Married	ref	0.83(0.77,0.89)	<0.001	
Never married	ref	0.88(0.80,0.96)	0.005	
Divorced	ref	0.94(0.79,1.12)	0.48	
PIR				0.82
≤1.30	ref	0.86(0.79,0.92)	<0.001	
1.31–3.49	ref	0.83(0.75,0.91)	<0.001	
≥3.50	ref	0.85(0.77,0.95)	0.003	
Smoking				0.31
No	ref	0.84(0.77,0.90)	<0.001	
Yes	ref	0.88(0.81,0.95)	0.003	
Drinking				0.28
No	ref	0.80(0.70,0.92)	0.003	
Yes	ref	0.86(0.81,0.91)	<0.001	
Regular period				0.61
No	ref	0.82(0.71,0.96)	0.01	
Yes	ref	0.86(0.80,0.91)	<0.001	
PID				0.75
No	ref	0.85(0.80,0.91)	<0.001	
Yes	ref	0.87(0.75,1.02)	0.09	
DM				0.29
No	ref	0.84(0.78,0.90)	<0.001	
Yes	ref	0.90(0.79,1.04)	0.14	

eGDR, estimated glucose disposal rate; BMI, body mass index; PIR, poverty income ratio; PID, pelvic inflammatory disease; DM, diabetes mellitus. ***P value<0.001.

### 3.4 Sensitive analysis on the relationship between eGDR and infertility

To further validate the robustness of the findings, a sensitivity analysis using unweighted logistic regression was executed. This additional analysis underscored a statistically significant relationship between eGDR and infertility incidence. Increased eGDR levels were associated with a reduced likelihood of experiencing infertility (OR = 0.89, 95% CI 0.84-0.93) (**Table 4**).

**Table 4:** Unweighted logistic regression analysis of HOMA-IR and eGDR in relation to infertility.

	Model 1		Model 2		Model 3	
	OR (95% CI)	*P* value	OR (95% CI)	*P* value	OR (95% CI)	*P* value
HOMA-IR	1.02(0.99,1.05)	0.2	1.02(0.98,1.05)	0.26	1.02(0.98,1.06)	0.35
Continuous eGDR	0.87 (0.84,0.91)	<0.001***	0.89 (0.86,0.93)	<0.001***	0.89 (0.84,0.93)	<0.001**
eGDR-Q1	Reference	–	Reference	–	Reference	–
eGDR-Q2	0.66 (0.49,0.89)	0.01*	0.71 (0.53,0.96)	0.03*	0.71 (0.52,0.97)	0.03*
eGDR-Q3	0.49 (0.36,0.67)	<0.001***	0.54 (0.39,0.74)	<0.001***	0.53 (0.38,0.74)	<0.001***
eGDR-Q4	0.34 (0.24,0.48)	<0.001***	0.40 (0.28,0.58)	<0.001***	0.38 (0.26,0.56)	<0.001***
*P* for trend		<0.001***		<0.001***		<0.001***

Data are presented as OR (95% CI). Model 1 remained unadjusted. Model 2 adjusted for age, race/ethnicity and education levels. Model 3 adjusted for age, race/ethnicity, education levels, poverty income ratio, marital status, smoking, drinking, hemoglobin, TC, DM, regular period and PID, adrenocortical insufficiency and sex hormone dysfunction. ***P value<0.001, **P value<0.01, *P value<0.05.

### 3.5 Comparison of HOMA-IR and eGDR in predicting infertility

**Figure 4:**
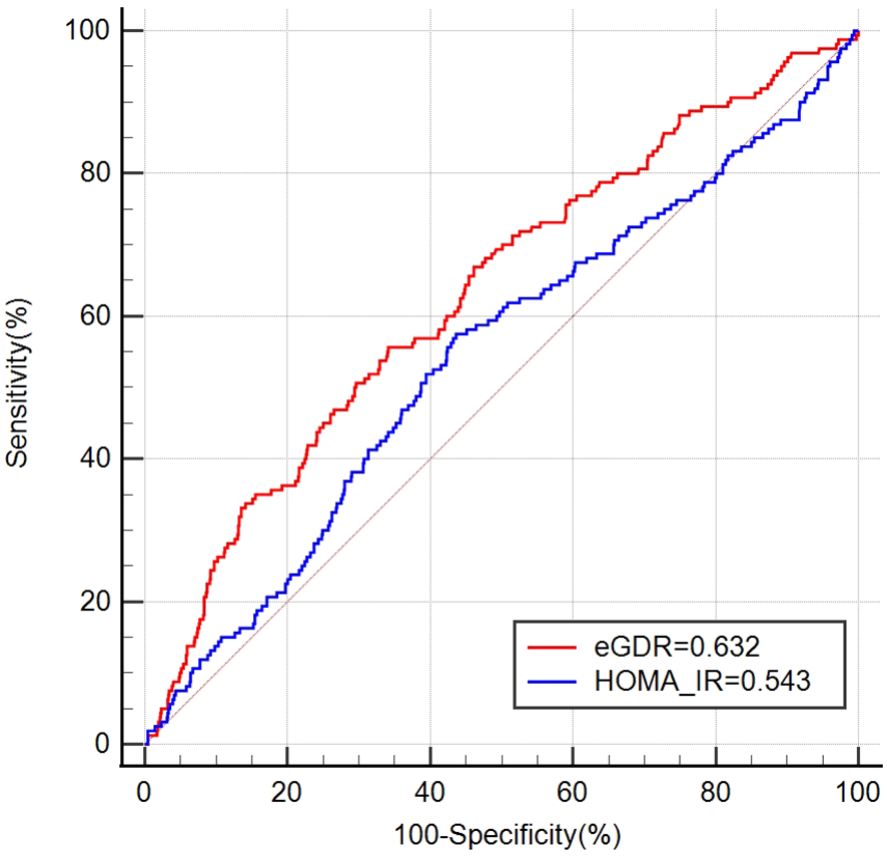
ROC curves for HOMA-IR and eGDR to predict infertility.

The ROC curve results indicate that the AUCs for HOMA-IR and eGDR in predicting infertility were 0.543 (95% CI: 0.514, 0.572) and 0.632 (95% CI: 0.603, 0.660), respectively (**Figure 4**). This shows that eGDR significantly outperformed HOMA-IR in predicting infertility (*P* < 0.001).

## 4 Discussion

This study, conducted as a cross-sectional analysis, explored the relationship between eGDR and infertility in women, utilizing data from the NHANES database. The study revealed a decreasing prevalence of female infertility with increasing eGDR levels. Subgroup analysis indicated that age played a role in modifying the relationship between eGDR and infertility. The RCS analysis demonstrated a nonlinear inverse association between eGDR and female infertility. The ROC curve suggested that eGDR was significantly better than HOMA-IR in predicting infertility.

As far as we are aware, this study is the inaugural exploration of the correlation between eGDR and female infertility. Originally designed as a clinical indicator for assessing insulin resistance in diabetic patients, eGDR was utilized in this context (24–26). The formula for eGDR includes WC, HP, and HbA1c, and is easy to obtain in the clinic. Unlike HIEC, eGDR’s simplicity and user-friendly nature make it a valuable clinical tool, offering a reliable assessment of IR. In addition to predicting IR, earlier investigations have indicated that, hat lower eGDR levels can predict diabetic peripheral neuropathy (27), renal deterioration in patients with type 2 diabetes mellitus (28), risk of incident cardiovascular diseases in non-diabetic individuals (29), as well as functional outcomes in acute ischemic stroke patients (14). The robust association of eGDR with a spectrum of diseases underscores its robust functionality as an IR biomarker.

Prior research has established that IR significantly impacts female reproductive function (7, 30, 31). Furthermore, PCOS is a common endocrine disorder affecting women during their reproductive years. Individuals diagnosed with PCOS commonly present with IR and hyperinsulinemia (32). IR not only induces hyperinsulinemia but also directly impacts ovarian function, exacerbating the reproductive irregularities associated with PCOS and ultimately contributing to infertility (33, 34). What’s more, IR can influence outcomes in assisted reproduction among infertile women. In a prospective cohort study involving non-obese women without PCOS, IR was found to correlate with a reduced proportion of mature eggs and diminished embryo quality (8). Another retrospective analysis, encompassing 329 non-obese women undergoing *in vitro* fertilization-embryo transfer, revealed that elevated homeostasis model assessment of IR levels were associated with reduced clinical pregnancy rates, independent of the presence of comorbid PCOS (35). In this study, each one-unit increase in eGDR was associated with a 14% decrease in the prevalence of self-reported female infertility. Furthermore, this inverse association was only influenced by the age of the participants. It may due to women’s fertility is significantly influenced by age, as advancing years coincide with reproductive senescence, diminished ovarian reserve, and a deterioration in oocyte quality (36). These factors collectively contribute to the potential for infertility.

HOMA-IR is an index based on fasting plasma insulin and glucose concentrations, primarily reflecting hepatic IR, and it may be influenced by insulin levels and the use of insulin-sensitizing agents (37, 38). In contrast, eGDR is a readily obtainable clinical measure that incorporates hypertension, a condition closely associated with skeletal muscle IR (37, 39). High levels of eGDR indicate better insulin sensitivity (17, 40, 41), and in this study, eGDR showed a negative correlation with female infertility, suggesting that IR, which leads to hyperinsulinemia, negatively impacts reproductive health. By integrating these factors, eGDR provides a more comprehensive assessment of insulin sensitivity. Overall, eGDR may better predict reproductive health outcomes by capturing the interplay between blood sugar control, body fat distribution, and metabolic conditions such as hypertension. We evaluated the predictive efficiency of eGDR compared to HOMA-IR for infertility and found that eGDR demonstrated superior predictive value. This indicates that eGDR has significant potential as a predictive marker for female infertility. However, it is important to note that this study is based on cross-sectional data from the NHANES database. Therefore, we recommend that future prospective studies be conducted to further validate our findings. Only after such validation should eGDR be considered for routine evaluation in clinical practice.

IR adversely impacts female fertility through several mechanisms. Firstly, in patients with PCOS, IR induces hyperinsulinemia, affects follicular membrane cells, synergizes with luteinizing hormone to increase androgen production, and disrupts the synthesis of sex hormone-binding globulin. This cascade results in elevated levels of bioactive androgens, ultimately impairing ovulation (42, 43). Secondly, IR damages telomeres and spindles through oxidative stress, which leads to abnormalities during oocyte meiosis, failing embryo implantation, or miscarriage (7, 44). Thirdly, during oocyte maturation, exposure to IR results in heightened production of reactive oxygen species. This increase diminishes mitochondrial enzyme activity and lowers antioxidant capacity, thereby impairing mitochondrial function. Consequently, oocyte quality is compromised and follicle depletion occurs due to these detrimental effects of IR (20, 45). IR also impacts endometrial tolerance by suppressing endometrial metaplasia through the modulation of AMPK and PI3K-Akt pathways, as well as influencing glucose metabolism (46, 47).

This study exhibits notable strengths. Firstly, it draws upon data sourced from NHANES, known for its representative nature achieved through robust sampling methodologies and weighted statistics. Secondly, the study underscores the stability and reliability of the association between eGDR and infertility by incorporating pertinent covariates, conducting sensitivity analyses, and exploring subgroup dynamics. However, several limitations warrant consideration. Firstly, due to its cross-sectional design, the study is unable to determine causal relationships between variables, necessitating prospective investigations for further validation. Secondly, due to the reliance on self-reported questionnaires for defining infertility, the specific causes of female infertility remain unclear in the NHANES database, and the potential presence of infertility in male partners was not examined. Nonetheless, prior studies have demonstrated that self-reported assessments can still yield valuable scientific insights (48–51). Thirdly, despite the comprehensive inclusion of numerous covariates, potential confounding variables influencing the eGDR-infertility relationship may not have been fully accounted for, such as uterine dysfunction, ovarian tumors and congenital adrenal hyperplasia, and the amount of data may not be comprehensive due to the limited scope of the NHANES database investigation. This underscores the need for future prospective studies that specifically include hormonal evaluations as covariates. Finally, while the findings are statistically representative post-sampling and weighted analyses, their generalizability is restricted solely to the American population and cannot be extrapolated universally. And as a cross-sectional study, our sample size is not particularly large. Therefore, we hope to conduct related research in different populations (such as European or Chinese cohorts) in the future, particularly large-scale prospective studies, to address this limitation.

## 5 Conclusion

In conclusion, this study suggests an association between increased eGDR levels and a decreased incidence of infertility among women. These findings underscore the significance of managing IR in safeguarding female reproductive health. Future research should include more comprehensive prospective studies to validate the findings of this investigation.

## Data Availability

The datasets presented in this study can be found in online repositories. The names of the repository/repositories and accession number(s) can be found below: https://www.cdc.gov/nchs/nhanes/.
