# Supplementary material for: Association between estimated glucose disposal rate and female infertility: a cross-sectional study
Source: Front Endocrinol (Lausanne). 2024 Nov 12;15:1474738. doi: 10.3389/fendo.2024.1474738 (PMC11588443; doi:10.3389/fendo.2024.1474738)
Supplement: Supplementary file 1 [file Table1.docx]

**Table S1. Clinical characteristics grouped by eGDR quartiles.**

| Variables | Overall | eGDR-Q1 | eGDR-Q2 | eGDR-Q3 | eGDR-Q4 | *P* value |
| --- | --- | --- | --- | --- | --- | --- |
| Age, years | 32.52±0.23 | 35.12±0.28 | 33.27±0.42 | 32.39±0.40 | 29.68±0.41 | <0.001*** |
| Race/ethnicity, % |  |  |  |  |  | <0.001*** |
| White | 58.31 (49.96,66.66) | 53.31 (47.06,59.56) | 54.80 (47.42,62.18) | 59.94 (53.62,66.25) | 64.32 (59.44,69.19) |  |
| Black | 12.47 (10.07,14.88) | 20.40 (15.64,25.15) | 12.33 ( 8.36,16.29) | 9.18 (6.33,12.02) | 9.11 ( 6.76,11.47) |  |
| Mexican | 11.30 ( 8.51,14.09) | 12.72 ( 8.92,16.52) | 14.83 (10.15,19.51) | 12.83 (8.95,16.71) | 5.14 ( 3.13, 7.16) |  |
| Others | 17.91 (15.70,20.13) | 13.57 (10.91,16.23) | 18.04 (13.98,22.10) | 18.05 (13.76,22.34) | 21.43 (17.82,25.03) |  |
| Education levels, % |  |  |  |  |  | <0.001*** |
| Less than high school | 9.84 ( 8.26,11.42) | 13.22 (10.46,15.98) | 11.67 ( 9.07,14.26) | 8.92 (6.23,11.61) | 6.12 (4.37, 7.88) |  |
| High school or equivalent | 18.30 (15.65,20.95) | 19.95 (15.70,24.21) | 23.25 (18.32,28.18) | 14.48 (10.82,18.13) | 16.12 (12.81,19.43) |  |
| College or above | 71.86 (64.32,79.39) | 66.82 (62.29,71.35) | 65.08 (59.40,70.76) | 76.61 (71.18,82.04) | 77.76 (73.41,82.10) |  |
| PIR, % |  |  |  |  |  | <0.001*** |
| ≤1.30 | 27.68 (25.21,30.15) | 33.65 (27.93,39.38) | 30.86 (25.80,35.92) | 25.56 (21.98,29.14) | 21.66 (18.22,25.11) |  |
| 1.31–3.49 | 36.88 (33.00,40.76) | 39.40 (34.25,44.55) | 40.39 (35.35,45.44) | 36.66 (32.34,40.98) | 31.58 (26.96,36.19) |  |
| ≥3.50 | 35.44 (30.03,40.84) | 26.94 (22.25,31.64) | 28.74 (23.24,34.25) | 37.78 (32.27,43.29) | 46.76 (41.60,51.92) |  |
| Marital status, % |  |  |  |  |  | <0.001*** |
| Married | 10.53 ( 8.57,12.49) | 14.83 (11.93,17.74) | 13.31 (9.43,17.19) | 10.32 (7.21,13.43) | 4.36 (2.97, 5.76) |  |
| Never married | 29.74 (26.18,33.29) | 23.63 (19.99,27.27) | 25.73 (21.26,30.21) | 27.65 (22.81,32.50) | 41.00 (35.44,46.55) |  |
| Divorced | 59.73 (53.70,65.77) | 61.54 (56.94,66.14) | 60.95 (55.37,66.54) | 62.03 (57.31,66.75) | 54.64 (48.88,60.41) |  |
| BMI, kg/m^2^ | 29.37±0.26 | 38.56±0.38 | 32.95±0.21 | 26.04±0.17 | 21.39±0.11 | <0.001*** |
| Hemoglobin, g/dl | 13.26±0.04 | 13.20±0.06 | 13.22±0.06 | 13.27±0.05 | 13.33±0.06 | 0.37 |
| waist circumference, cm | 95.72±0.59 | 117.38±0.83 | 104.19±0.40 | 88.70±0.22 | 76.08±0.28 | <0.001*** |
| HbA1c, % | 5.34±0.01 | 5.78±0.04 | 5.37±0.02 | 5.20±0.01 | 5.09±0.01 | <0.001*** |
| HOMA-IR | 3.19±0.12 | 5.56±0.28 | 3.98±0.34 | 2.01±0.09 | 1.43±0.06 | <0.001*** |
| TC, mmol/L | 4.66±0.03 | 4.85±0.06 | 4.77±0.05 | 4.61±0.04 | 4.44±0.04 | <0.001*** |
| Smoking, % |  |  |  |  |  | 0.004** |
| No | 67.57 (62.12,73.02) | 59.38 (54.56,64.20) | 68.58 (64.35,72.82) | 70.30 (65.80,74.80) | 70.94 (65.33,76.55) |  |
| Yes | 32.43 (28.38,36.48) | 40.62 (35.80,45.44) | 31.42 (27.18,35.65) | 29.70 (25.20,34.20) | 29.06 (23.45,34.67) |  |
| Drinking, % |  |  |  |  |  | 0.03* |
| No | 17.33 (13.96,20.70) | 17.90 (13.95,21.85) | 21.23 (16.35,26.11) | 16.42 (12.25,20.58) | 14.08 (10.15,18.01) |  |
| Yes | 82.67 (75.36,89.98) | 82.10 (78.15,86.05) | 78.77 (73.89,83.65) | 83.58 (79.42,87.75) | 85.92 (81.99,89.85) |  |
| DM, % |  |  |  |  |  | <0.001*** |
| No | 93.37 (85.70,101.04) | 79.12 (75.78,82.46) | 94.18 (92.45,95.90) | 98.65 (97.74,99.57) | 99.56 (99.19,99.94) |  |
| Yes | 6.63 (5.56, 7.70) | 20.88 (17.54,24.22) | 5.82 ( 4.10, 7.55) | 1.35 (0.43, 2.26) | 0.44 ( 0.06, 0.81) |  |
| Regular period, % |  |  |  |  |  | <0.001*** |
| No | 11.69 ( 9.91,13.48) | 16.24 (13.12,19.36) | 13.21 (10.23,16.18) | 11.40 ( 8.18,14.63) | 6.59 ( 4.24, 8.95) |  |
| Yes | 88.31 (81.32,95.30) | 83.76 (80.64,86.88) | 86.79 (83.82,89.77) | 88.60 (85.37,91.82) | 93.41 (91.05,95.76) |  |
| PID, % |  |  |  |  |  | <0.001*** |
| No | 95.51 (87.83,103.20) | 91.81 (88.87,94.76) | 96.11 (94.21,98.02) | 97.19 (95.75,98.62) | 96.44 (94.87,98.01) |  |
| Yes | 4.49 ( 3.35, 5.62) | 8.19 (5.24,11.13) | 3.89 (1.98, 5.79) | 2.81 (1.38, 4.25) | 3.56 (1.99, 5.13) |  |
| Adrenocortical insufficiency, % |  |  |  |  |  | 0.71 |
| No | 99.98 (92.10,107.86) | 100.00 (100.00,100.00) | 99.90 ( 99.72,100.09) | 100.00 (100.00,100.00) | 100.00 (100.00,100.00) |  |
| Yes | 0.02 (0.01, 0.07) | 0.00 ( 0.00,0.00) | 0.10 (0.01,0.28) | 0.00 ( 0.00,0.00) | 0.00 ( 0.00,0.00) |  |
| Sex hormonal dysfunctions, % |  |  |  |  |  | 0.39 |
| No | 99.82 (91.95,107.68) | 99.69 ( 99.26,100.12) | 100.00 (100.00,100.00) | 99.57 ( 98.95,100.19) | 100.00 (100.00,100.00) |  |
| Yes | 0.18 (0.01, 0.38) | 0.31 (0.01,0.74) | 0.00 ( 0.00,0.00) | 0.43 (0.02,1.05) | 0.00 ( 0.00,0.00) |  |
| Infertility, % |  |  |  |  |  | <0.001*** |
| No | 85.73 (79.19,92.27) | 77.26 (72.01,82.50) | 84.51 (81.23,87.80) | 86.83 (82.94,90.72) | 93.14 (90.84,95.43) |  |
| Yes | 14.27 (11.79,16.75) | 22.74 (17.50,27.99) | 15.49 (12.20,18.77) | 13.17 (9.28,17.06) | 6.86 (4.57, 9.16) |  |

Continuous data were presented as the mean±SEM, category data were presented as the proportion and 95% confidence interval. SEM, Standard Error of the Mean; eGDR, estimated glucose disposal rate; PIR, poverty income ratio; BMI, body mass index; HbA1c, glycosylated hemoglobin; HOMA-IR, homeostasis model assessment of insulin resistance; TC, total cholesterol; DM, diabetes mellitus; PID, pelvic inflammatory disease; *** *P* value<0.001, ** *P* value<0.01, * *P* value<0.05.
